# Periprosthetic Humeral Fractures After Short-Stem Reverse Shoulder Arthroplasty: Treatment Patterns, Classification, and Clinical Outcomes

**DOI:** 10.3390/jcm15010298

**Published:** 2025-12-30

**Authors:** Naoya Kubota, Katsumasa Nakazawa, Tomoya Manaka, Yoichi Ito, Yoshihiro Hirakawa, Ayako Ogura, Hidetomi Terai

**Affiliations:** 1 Saiseikai Senri Hospital, 1-1-6 Tsukumodai Suita-shi, Osaka 565-0862, Japan; 2 Department of Orthopedic Surgery, Graduate School of Medicine, Osaka Metropolitan University, 1-4-3 Asahimachi, Abeno-ku, Osaka 545-8585, Japan; 3 Ito Clinic, Osaka Shoulder Center, 1-10-12 Ueda Matsubara, Osaka 580-0016, Japan; 4 Ishikiriseiki Hospital, 18-28 Yayoicho Higashiosaka, Osaka 579-8026, Japan

**Keywords:** reverse total shoulder arthroplasty, periprosthetic humeral fracture, periprosthetic fractures, postoperative complications, short stem

## Abstract

**Background/Objectives:** Periprosthetic humeral fractures (PF) after reverse total shoulder arthroplasty (RSA) are expected to increase. This study investigated PF after RSA with short stems and reported outcomes. **Methods:** A total of 165 patients underwent short-stem RSAs between 2014 and 2023. Among them, patients who developed postoperative PFs were identified and classified by fracture location and stem loosening. Operative data, complications, and bone union time were analyzed. Clinical outcomes before injury and at final follow-up were evaluated. **Results:** PF occurred in 5/165 patients (3.0%). Based on our classification, four had type B1 fractures and one had a type B3 fracture. All underwent revision RSA (Re-RSA) with conversion to long-stem implants. Bone union was achieved in four patients, while one patient experienced infection without union. Among the four patients without complications, mean shoulder flexion declined from 138° pre-injury to 103°, abduction from 118° to 95°, external rotation from 37° to 31°, the American Shoulder and Elbow Surgeons (ASES) score from 82.0 to 68.7, Constant Score from 67 to 43, while the Visual Analog Scale (VAS) pain score increased from 1.7 to 2.6. **Conclusions:** All five cases of PF following short-stem RSA were stem-level (type B) fractures. All patients underwent Re-RSA using long-stem conversion. Four patients had bone union. Clinical outcomes at one year postoperatively had deteriorated mildly compared to pre-fracture. However, this change was not statistically significant. One patient had a postoperative infection, and bone union was not observed. This study indicates the need for caution regarding postoperative infections after RSA.

## 1. Introduction

Reverse total shoulder arthroplasty (RSA) was first reported by Paul Grammont et al. [1] in 1987. RSA is considered to be a good indication for cuff tear arthropathy, massive rotator cuff tears, and proximal humeral fractures. However, a high complication rate of approximately 40% has been reported worldwide, including infections, postoperative fractures, loosening, and instability [2,3,4]. Postoperative periprosthetic humeral fractures (PFs) have so far been considered a relatively rare complication, but they are becoming more common with the recent increase in incidence of RSAs being performed.

Incidence of PFs range from 0.9 to 2.0% of postoperative complications after RSA, and are more common in elderly women, suggesting an association with osteoporosis [5,6,7,8]. Therefore, PF is an issue of particular concern associated with RSA [9].

There are several classifications of PF, but the Wright and Cofield system, reported in 1995, is the most commonly used [10]. This classification defines Type A as fractures that are centered at the tip of the prosthesis and extend proximally more than one-third of the length of the stem. Type B fractures are centered at the tip of the prosthesis but with only slight proximal extension, while Type C fractures are distal to the tip of the stem. Although reports on treatment outcomes stratified by the Wright and Cofield classification are still limited, the effectiveness of Open Reduction and Internal Fixation (ORIF) for Type B PFs has been reported [5].

The Wright and Cofield classification focused on total shoulder arthroplasty (TSA) and long stem cases. In recent years, with the development of short stems, good outcomes have been reported [8,11]. However, new complications such as stress shielding have also been observed [12,13]. Furthermore, while there have been many reports of PFs following RSAs using short stems [14], few studies have evaluated and classified them.

Therefore, the purpose of this study was to investigate PFs following short-stem RSAs, develop a system of classification for short-stem PFs, and report treatment outcomes. Our hypothesis was that the types of PFs following short-stem RSAs are more common at the distal stem level, and that revision-RSAs (Re-RSAs) are likely to provide reasonable clinical outcomes. The proposed classification system for short-stem PFs is intended to be used to guide the treatment PF of short-stem RSAs.

## 2. Materials and Methods

This study is a retrospective multicenter case series nested within a short-stem RSA cohort. We recruited 413 patients who underwent RSA using short stems between 2014 and 2023 at our associated hospitals. Of these, 244 patients were excluded because they had insufficient clinical data or a follow-up duration of less than two years. A total of 169 patients with adequate data and at least two years of postoperative follow-up were included. Of these, four patients were excluded due to intraoperative fractures. Consequently, a total of 165 patients who underwent RSA using short stems were analyzed and we investigated cases that had PF after primary RSA retrospectively.

All primary RSAs were performed using the Ascend Flex implant (Tornier-Stryker, Kalamazoo, MI, USA) by deltopectoral approach. Cemented stems were used in six cases, while cementless stems were used in the remaining 159 cases. The standard postoperative rehabilitation protocol consisted of sling immobilization for two to three weeks. Passive ROM exercises were initiated on the second postoperative day. Active ROM exercises were permitted without restrictions starting at six weeks postoperatively. Weight-bearing with loads of less than 1 kg was commenced at six weeks, followed by a progressive increase thereafter.

**Figure 1:**
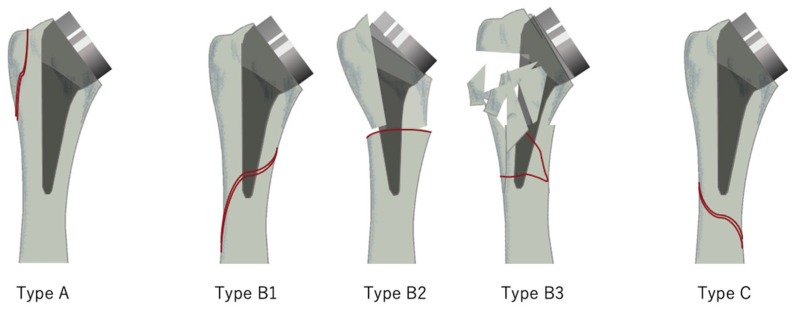
Classification of PFs (periprosthetic humeral fractures) in this study. Type A involves the lesser or greater tuberosity. Type B1 is at stem level with stem stability. Type B2 is at stem level without stem stability. Type B3 is at stem level without stem stability nor bone stock. Type C originates below the tip of the stem. At stem level means the fracture line overlaps the implant.

PF patterns were reviewed using the classification based on the system originally developed in this study. Fractures proximal to the stem were classified as type A, those at the stem level as type B, and those distal to the stem as type C. Fractures with no stem loosening were further classified as B1, those with loosening as B2, and those with bone loss as B3 (Figure 1).

In this study, “stem loosening” was defined as the presence of a radiolucent line around the implant, which represents implant migration or alignment change on plain radiographs. Intraoperative evaluation of loosening in cementless stems was defined as loss of osseointegration between the implant and the humerus. For cemented stems, loosening was considered present when failure was observed at the cement-bone interface or the cement-implant interface. However, in this study, Re-RSA was performed regardless of the presence or absence of loosening. “At the stem level” indicates that the fracture line overlaps with the stem, irrespective of the stem length. “Bone loss” refers to comminution at the fracture site resulting in a bone defect.

Two surgeons (certificated doctors of orthopedics for more than ten years and specializing in shoulder joint surgery, K.N and T.M) performed the analysis twice, one month apart, to obtain the intra-observer and inter-observer κ values for the system of classification developed in this study. The indications for surgery for PF and the surgical procedure were comprehensively determined by a shoulder surgeon (T. M.) based on fracture classification, degree of dislocation, stem loosening, and bone defects. Operative time and blood loss were also investigated by the anesthesia records.

In addition, the presence of bone union and complications were investigated in each case. Clinical outcomes of pre-fracture and postoperatively were also investigated. Pre-fracture clinical outcomes were defined as the most recent follow-up data obtained after the primary RSA and before the PF occurred. The endpoint for clinical outcomes was assessed at one year following Re-RSA. The American Shoulder and Elbow Surgeons (ASES) scores, pain Visual Analog Scale (VAS) scores, range of motion, including active flexion, abduction, and external rotation, and Constant Score (CS) were evaluated as clinical outcomes. Bone union and complications were assessed from one month to one year postoperatively. Bone union was assessed using four radiographic views: standard anteroposterior, Grashey anteroposterior, scapula-Y, and axillary images. Bone union was defined as the presence of a bridging bone on radiographs or the disappearance of fracture lines on two or more radiographs at different projection angles [15].

### Statistical Analysis

The averages and standard deviations for each parameter were calculated using data from the first and second acquisition sessions. Statistical analysis was performed using the exact Wilcoxon signed-rank test. The paired effect size was estimated using the Hodges–Lehmann median difference, presented with its corresponding 95% confidence interval. Statistical significance was defined as *p* < 0.05. R software, version 4.2.3 (R Foundation, Vienna, Austria) was used for the analysis.

Fracture classifications were used to interpret the intra-observer and inter-observer κ value: values between 0.00 and 0.20 indicate slight concordance, values between 0.21 and 0.40 indicate mild concordance, values between 0.41 and 0.60 indicate moderate concordance and values between 0.61 and 0.80 indicate substantial concordance, while values ≥ 0.80 indicate almost perfect concordance. Due to the small number of cases, the κ value obtained in this study does not substantiate its reliability and is presented solely as a reference value.

## 3. Results

The indications for primary RSA are shown in Table 1.

**Figure 2:**
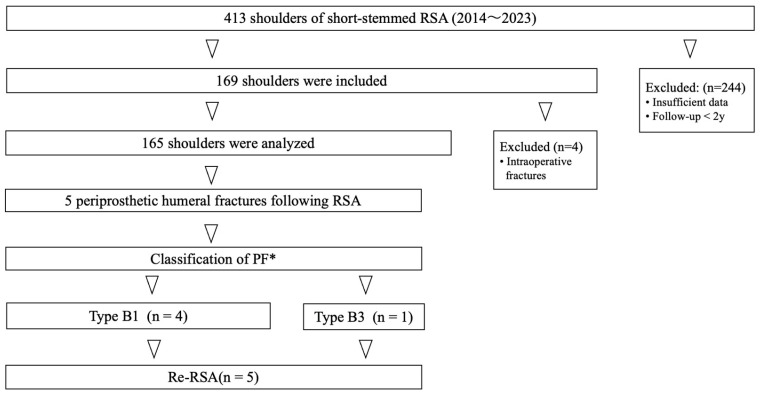
Classification of PFs for short-stem RSAs created in this study. *PF, periprosthetic humeral fractures.

PF after short-stem RSA occurred in 5/165 patients, representing 3.0% of all cases. The average age of these five patients was 72.4 years (range: 47–87 years) and the cohort included two males and three females. Mean time from the first surgery to PF injury was 657.6 days (range: 49–1335 days) (Table 2). These five patients were identified from the database and investigated retrospectively (Figure 2). All five patients were available for follow-up and radiographic assessment for more than one year after PF.

**Figure 3:**
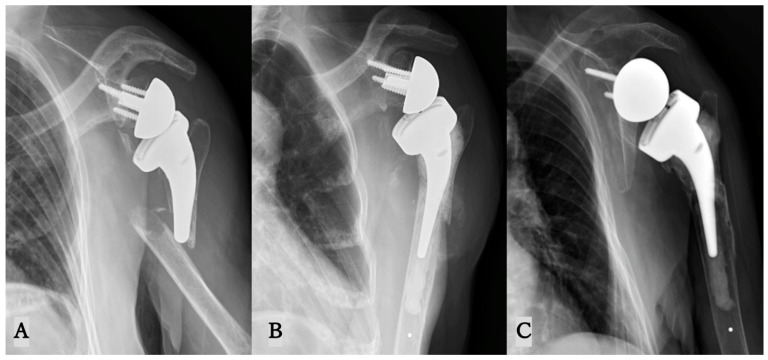
Re-RSA using long-stem conversion and cement fixation. (**A**): PF of type B1 following RSA. (**B**): RSA with cemented long stem. (**C**): Union can be observed on X-ray at the last follow up. PF, periprosthetic humeral fracture. RSA, reverse total shoulder arthroplasty.

**Figure 4:**
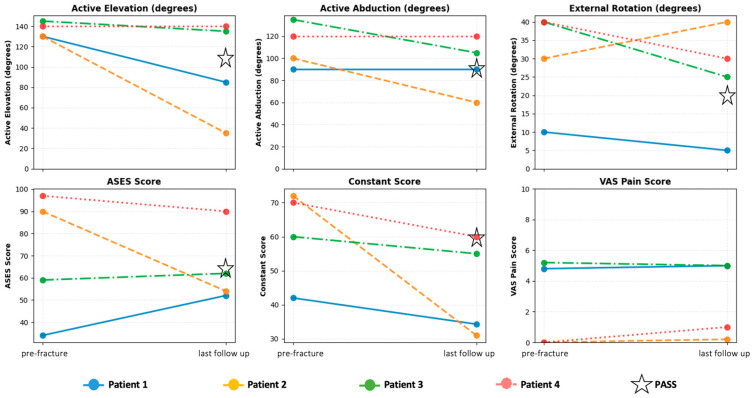
Spaghetti plots of clinical outcomes of this study and PASS. ASES score, American Shoulder and Elbow Surgeons score. PASS, Patient acceptable symptomatic state as reported by K.A. Hao et al. [16]. VAS pain score, Visual Analog Scale pain score.

Four had type B1 fractures and one had a type B3 fracture, according to our PF classification. The intra-observer κ value of the classification of PF was 1.0 at the first measurement and 0.63 at the second measurement, while the inter-observer κ values were 0.59 and 0.59, respectively (Table 3). All five patients underwent Re-RSA, which were performed with a deltopectoral approach using long-stem conversion and cement fixation (Figure 3). For type B3 fractures, additional intraoperative wiring was performed. Mean operative time was 106 min (69–144 min) and mean blood loss was 214 g (range, 110–420 g). The postoperative rehabilitation protocol for Re-RSA was identical to that used for primary RSA. Bone union was observed in four patients, with an average time to union of 92 days (45–356 days). In contrast, one patient had a postoperative infection, and bone union was not observed. Clinical results were obtained for the four patients who had no postoperative complications. At the time of pre-fracture and postoperative follow-up, clinical outcomes changed as shown in Figure 4 and Table 4. Clinical outcomes at one year postoperatively had mildly deteriorated compared to pre-fracture. However, this change was not statistically significant (Table 5). When compared to the established Patient Acceptable Symptomatic State (PASS) thresholds following Re-RSA, some patients in our series achieved this benchmark, while others did not (Figure 4).

### Complications

Postoperative infection occurred in one patient with Re-RSA. The patient had no notable past medical history except for high BMI. The surgical parameters for the Re-RSA were 85 min in duration and 160 g in blood loss, with an interval of 48 days from the primary RSA. Periprosthetic joint infection was detected at postoperative 6-month follow-up. Vancomycin was administered despite the absence of significant culture growth. Treatment consisted of implant removal and placement of a vancomycin-impregnated cement spacer. The patient’s management was complicated by a cerebellar infarction, necessitating transfer to another hospital.

## 4. Discussion

Of the 165 patients who underwent short-stem RSA, 5 patients had postoperative PF, resulting in an incidence rate of 3.0%. In this study, we classified postoperative PF following RSA, focusing on short-stem cases, and reported the clinical outcomes. Four patients had type B1 fractures, which are fractures without loosening at the stem level. The fifth patient who underwent Re-RSA for PF had an infection and bone union was not observed. However, all other cases showed bone union and postoperative clinical outcomes were statistically as good as the pre-injury outcome.

In 1995, Wright et al. [10] classified PF after shoulder arthroplasty according to the fracture site. According to this classification, type A fractures are those in which the fracture line extends more than one-third of the length of the stem proximally centered on the stem tip. Type B fractures are those in which the fracture line does not extend proximally centered on the stem tip. Finally, type C fractures were classified as those distal to the stem tip. In 1999, Worland et al. [17] modified this classification as follows: type B1 fractures are spiral fractures with stable stems, type B2 fractures are transverse or short oblique fractures near the stem tip with stable stems, and type B3 fractures have unstable stems. These classifications are mainly used with reference to TSA. In addition, because such studies mainly recruited long-stem cases, classification may have been difficult for short-stem cases, whose incidence has been increasing in recent years. In fact, the intra-observer κ value in Wright and Cofield’s classification was reported to be 0.37 [18]. However, this classification method is not very objective for short-stem cases. In addition, Campbell et al. [19] classified PF based on the location of the fracture but did not consider stem loosening or bone loss. Although Gordon et al. [20] also proposed a relatively simple and clear classification, it was aimed at only hemiarthroplasty or TSA. In other words, there is currently no classification method for PF following RSA, especially for short stems. Our study is the first to classify PF following short-stem RSA. The classification system for PF developed in this study was based on the Vancouver classification of periprosthetic femoral fractures reported in 1995 [21,22]. The inter-rater reliability of this study was 0.59, which is a moderate result, while the intra-rater reliability was 1.0 for the first acquisition session and 0.63 for the second session, which is substantial. Therefore, we believe that our classification system is simple, reliable, and valid. However, it should be taken into consideration that in some cases it is difficult to assess whether the stem is loose or whether the fracture extends to the stem.

This study aimed to evaluate morphology of short-stem PF, using our system of classification. All cases were classified as type B, that is, stem-level fractures. In particular, fractures centered on the stem tip and extending distally to the tip were more common. We consider this to be related to previous reports, in which postoperative bone mineral density tended to decrease around the stem tip and distally from the stem tip after short-stem RSA [12,13,20,23].

Regarding the incidence rate of postoperative PF following RSA, a systematic review by Shah et al. [7] reported a rate of 1.2%. Another systematic review reported an incidence of 1.4% of postoperative [8], while Garcia-Fernandez et al. [15] in a study of 203 RSA cases reported 4 (2.0%) cases of PF. From these studies, the incidence of PF following RSA appears to be between 1.2 and 2.0%. These studies are subject to all RSAs, not only short stems. Regarding short-stem RSA, PF incidence rate in a study by Atoun E et al. [11] was 12.9%, which is higher than that which was seen in other studies. Similarly, the incidence rate of short-stem PF in our study was 3.0%, which was relatively higher. Short-stem designs have been shown to be associated with an increased risk of postoperative PF [13].

It has been suggested that the treatment of PF should be determined by stem loosening of either ORIF or Re-RSA [18,24]. In a biomechanical study of periprosthetic femoral fractures of Vancouver types B1 and B2, the stiffness and deformed shapes of six alternative fixation methods for periprosthetic femoral fractures at a load of 2300 N were examined [25]. This study revealed that the plating method was considerably less stiff than the long-stemmed revision method, which was performed regardless of stem loosening. This is because it is sometimes difficult to determine whether the stem is loose before surgery. Another reason is that Re-RSA is minimally invasive. Fixation with a plate requires extensive soft tissue dissection, which increases the invasiveness of the procedure. However, stem removal can sometimes be difficult in Re-RSA, leading to iatrogenic fractures. An estimated 81% of intraoperative fractures are reported to have occurred during removal of the humeral component [26]. However, this may not necessarily apply to short-stem RSA. We did not have any difficulty removing the stem intraoperatively in any of the short-stem cases. This is demonstrated by the fact that the time required for Re-RSA surgery was not long in our hospital.

Regarding treatment of PF following RSA, García-Fernández et al. [5] reported good results of ORIF for Cofield classification type B. However, this study assessed only bone union and complications, and clinical outcomes such as range of motion were not reviewed. Although Tansey et al. [27] also reported better clinical outcomes with ORIF than Re-RSA in PF following RSA, we consider that there might have been a case selection bias in this regard. Additionally, these studies did not consider stem length. K.A. Hao et al. [16] reported patient acceptable symptomatic state (PASS) of clinical outcome scores and active ROM after Re-RSA. The PASS they reported were as follows: AE, 110°; active abduction, 98°; ER, 19°; ASES, 63.5; CS, 59.1. Compared to the results of this study, some cases achieved the defined PASS value while others did not. It is possible that the number of patients achieving the PASS threshold might increase with longer follow-up, as the referenced PASS value was defined at two years postoperatively, compared to the one-year endpoint of our study. Thus, Re-RSA can be considered to be an effective treatment option for PF after short-stem RSA. Further investigation with more cases is necessary. At the same time, it is necessary to keep the risk of postoperative infection in mind. *Propionibacterium acnes*, which is difficult to culture, accounts for 52% of such infections according to Bohsali et al. [9]. Tansey et al. [27] reported a similar PJI rate following surgery for periprosthetic shoulder fracture: 2.5% (1/40) after Re-RSA and 2.4% (1/41) after ORIF.

The first limitation of this study is that the follow-up rate was insufficient, and it is probable that not all cases were included. We included patients who could be followed up for more than at least two years. However, there may have been patients who had PF after the follow-up period. Second, the PF sample size was small. An increase in the number of cases is likely to lead to the identification of appropriate treatment strategies for each classification. Third, this was a retrospective study, which is likely to introduce case selection bias, particularly with regard to treatment modalities. Fourth, comparison groups for Re-RSA, such as ORIF, did not exist in our study. The surgical procedures were performed at institutions where shoulder surgeries were more frequently carried out than trauma surgeries, and where RSA was more readily adopted relative to ORIF. Furthermore, the surgeons were shoulder specialists with substantial experience in Re-RSA, which likely influenced the preference for this procedure. Due to the absence of cases treated with ORIF at these centers, a comparative analysis between the two techniques was not feasible, thereby precluding any definitive conclusions regarding the superiority of Re-RTSA. Fifth, the inter-observer reliability of the radiographic bone union criteria could not be adequately assessed owing to the limited number of cases. A future study with a larger cohort is warranted to establish a treatment algorithm based on the classification system used in this study.

## 5. Conclusions

Of the 165 patients who underwent short-stem RSA, 5 had postoperative PF with an incidence rate of 3.0%. We developed a clear classification system specifically for PF after short-stem RSA and these five patients were classified according to this system. In cases of PF after short-stem RSA, type B1 fractures without loosening at the stem level were the most common (4/5 cases). One patient who underwent Re-RSA for PF after short-stem RSA had an infection and did not achieve bone union. The remaining four patients achieved bone union, and while clinical outcomes at one year postoperatively had deteriorated mildly compared to pre-fracture, this change was not statistically significant. Therefore, Re-RSA appears to be an effective treatment option for Type B PF following short-stem RSA. However, this study, which is one of a small number of single-arm cohort studies, suggests a need for caution regarding postoperative infections after RSAs.

## Figures and Tables

**Table 1:** Indications for primary short-stem RSA.

Cuff tear arthropathy, n (%)	89 (53.9)
Massive rotator cuff tears, n (%)	61 (37.0)
Osteoarthritis, n (%)	9 (5.5)
Chronic dislocations, n (%)	4 (2.4)
Rheumatoid arthritis, n (%)	2(1.2)

RSA, reverse total shoulder arthroplasty.

**Table 2:** Demographic data of PF cases after short-stem RSA.

Patient	Gender/Age	BMI (kg/m^2^)	Time from Primary RSA to Fracture (Days)	Stem of Primary RSA	Classification of PF by This Study
1	Female/80	25	469	short/cementless	B3
2	Female/74	27	1335	short/cementless	B1
3	Male/74	25	730	short/cementless	B1
4	Female/87	23	705	short/cemented	B1
5	Male/47	29	49	short/cementless	B1

BMI, body mass index. GT, greater tuberosity. PF, periprosthetic humeral fractures. RSA, reverse total shoulder arthroplasty.

**Table 3:** Reliability of PF classification in this study.

Reliability Type	Mean κ-Value (Range)	
Inter-observer	0.59 (0.59–0.59)	Moderate agreement
Intra-observer	0.82 (0.63–1.0)	Almost perfect agreement

PF, periprosthetic humeral fracture. RSA, reverse total shoulder arthroplasty.

**Table 4:** Surgical and postoperative data of PF cases after short-stem RSA.

Patient	Classification of PF by This Study	Operation Time (min)	Blood Loss (g)	Time to Union (Day)	Complications
1	B3	120	200	356	-
2	B1	144	180	111	-
3	B1	69	110	45	-
4	B1	113	420	121	-
5	B1	85	160	nonunion	infection

PF, periprosthetic humeral fractures. RSA, reverse total shoulder arthroplasty.

**Table 5:** Changes in clinical outcomes from pre-fracture to last follow-up.

	Exact Wilcoxon Signed-Rank Test	Hodges–Lehmann Median Difference	95% CI for Median Difference
Active Elevation	W = 0 two-tailed *p* = 0.25	−36.25°	[−95, 0]
Active Abduction	W = 0 two-tailed *p* = 0.50	−17.5°	[−35, 0]
External Rotation	W = 2.5 two-tailed *p* > 0.50	−6.25°	[−12.5, +2.5]
ASES Score	W = 4 two-tailed *p* = 0.6875	−4.5	[−16.5, +10.5]
Constant Score	W = 0 two-tailed *p* = 0.125	−9.425	[−24.35, −7.5]
VAS Pain Score	W = 2 two-tailed *p* = 0.25	+0.2	[0, +0.6]

ASES score, American Shoulder and Elbow Surgeons score. VAS Pain score, Visual Analog Scale pain score. Calculated by Exact Wilcoxon signed-rank test. Statistically significant difference (*p* < 0.05).

## Data Availability

The datasets generated and analyzed during the current study are not publicly available due to restrictions. However, data may be made available to qualified researchers upon reasonable request and with approval from the institutional review board or ethics committee. Please contact the corresponding author for further information.
